# Early and long-term results of minimally invasive mitral valve surgery through a right mini-thoracotomy approach: a retrospective propensity-score matched analysis

**DOI:** 10.7717/peerj.4810

**Published:** 2018-05-28

**Authors:** Sabreen Mkalaluh, Marcin Szczechowicz, Bashar Dib, Anton Sabashnikov, Gabor Szabo, Matthias Karck, Alexander Weymann

**Affiliations:** 1 Department of Cardiac Surgery, Heart and Marfan Center—University of Heidelberg, Heidelberg, Germany; 2 Department of Cardiothoracic Surgery, Heart Center, University of Cologne, Cologne, Germany

**Keywords:** Anterolateral thoracotomy, Mitral regurgitation, Mitral valve surgery

## Abstract

**Background:**

Minimally invasive mitral valve surgery (MVS) via right mini-thoracotomy has recently attracted a lot of attention. Minimally invasive MVS shows postoperative results that are comparable to those of conventional MVS through the median sternotomy as per various earlier studies.

**Methods:**

Between 2000 and 2016, a total of 669 isolated mitral valve procedures for isolated mitral valve regurgitation were performed. A propensity score-matched analysis was generated for the elimination of the differences in relevant preoperative risk factors between the cohorts and included 227 patient pairs. Only degenerative mitral valve regurgitation was included. The aim of our study was to examine if the minimally MVS is superior to the conventional approach through sternotomy based on a retrospective propensity-matched analysis. The primary endpoints were early mortality and long-term survival. The secondary endpoints included postoperative complications.

**Results:**

The in-hospital mortality rate was significantly higher within the conventional sternotomy cohort (3.1%, *n* = 7 vs 0.4%, *n* = 1 for the minimally invasive cohort; *p* = 0.032). The incidence of stroke and exploration for bleeding was comparable. In contrast, the necessity for dialysis was significantly lower in the minimally invasive cohort (*p* = 0.044). Postoperative pain was not significantly lower in the minimally invasive MVS cohort (*p* = 0.862). While patients who underwent minimally invasive MVS experienced longer bypass and cross-clamp times, their lengths of stay in the intensive care unit and in the hospital, did not differ from the conventionally operated collective (*p* = 0.779 and *p* = 0.516), respectively. The mitral valve repair rate of 81.1% in the minimally invasive cohort was significantly superior to that of the conventional approach, which was 46.3% (*p* < 0.0001). The one-, five-, and 10-year survival rates were significantly higher in the minimally invasive cohort compared to the conventional approach (96%, 90%, and 84% vs. 89%, 85%, and 70%; log rank *p* = 0.004).

**Conclusion:**

Despite prolonged cardiopulmonary bypass and cross-clamping times, the minimally invasive MVS may be considered a safe approach that is equivalent to standard median sternotomy with lower early mortality and superior long-term survival.

## Introduction

Mitral valve disease is the most common valvular heart disorder, while mitral valve regurgitation is the most common pathology with a prevalence of 72% among all mitral valve disorders in industrialized countries (Bothe & Beyersdorf, 2016; Nishimura et al., 2016). Mitral valve repair is the gold standard for the treatment of degenerative mitral valve regurgitation (Badhwar et al., 2012; Mazine et al., 2015; Miceli et al., 2015).

Minimally invasive mitral valve procedures were introduced into clinical practice in the 1990s and represent a standard approach at specialized centers for mitral valve surgery (MVS). Various recent studies have demonstrated similar or superior outcomes after minimally invasive MVS compared with MVS through conventional median sternotomy (conv-MVS) (Atluri et al., 2016; Downs et al., 2016; Galloway et al., 2009; Walther, Falk & Mohr, 2004). Despite longer cardiopulmonary bypass (CPB) and cross-clamp times in mini-MVS, this approach led to comparable rates for early mortality, long-term survival and freedom from redo surgery (Atluri et al., 2016; Goldstone et al., 2013; Sundermann, Czerny & Falk, 2015). Furthermore, it was shown that in patients undergoing mini-MVS the occurrence of postoperative complications like mediastinal bleeding, acute renal failure, wound infection, atrial fibrillation, and pacemaker insertion was similar or lower than that in patients undergoing conv-MVS (Atluri et al., 2016; Cheng et al., 2011; Sundermann, Czerny & Falk, 2015).

The aim of this present study is to investigate the influence of the minimal invasive approach through lateral thoracotomy on outcome and survival. For that, we evaluated preoperative characteristics, operative data, postoperative events, and long-term survival in our patient collective undergoing isolated MVS due to mitral valve regurgitation from 2000 to 2016 in a non-high volume center for valve surgery. For comparing mini-MVS vs conv-MVS a propensity score-matched analysis was generated based on age, sex, co-morbidities, and preoperative clinical data. The clinical outcomes were investigated.

## Methods

### Study population

During the period from 2000 to 2016, a total of 669 isolated mitral valve procedures for isolated mitral valve regurgitation were recorded, among them 317 (47.4%) with a minimally invasive approach through right mini-thoracotomy. Degenerative mitral valve disease was the cause of mitral insufficiency in our matched cohort. The patients were divided into two groups: those who underwent mini-MVS and those who underwent conv-MVS via standard sternotomy; 227 pairs were generated.

The matched groups were similar with regard to selected preoperative co-morbidities, and the haemodynamic and demographic categories. All patients undergoing concomitant procedures were excluded. Furthermore, we considered the causes of mitral valve regurgitation; consequently, there was no statistical difference between the cohorts. Postoperative outcomes and major complications such as death, stroke, wound infection, reoperation for bleeding, re-intubation, blood product use, renal failure, atrial fibrillation, hospital and ICU lengths of stay, and survival were analyzed. This study was approved by the local ethics committee of the University of Heidelberg, medical faculty (approval no.: S-516/2016). Furthermore, this study follows the ICMJE Privacy and confidentiality guidelines. The participant consent and follow-up data were obtained by letter correspondence and clinical visits.

### Definitions

Early mortality was defined as death within 30 days after surgery or before discharge from the hospital. Postoperative respiratory failure was defined as re-intubation or tracheotomy. Postoperative stroke was defined as a new and permanent neurological disability or deficit. Postoperative temporary renal failure was defined as new necessity for dialysis during the hospital stay and as permanent renal failure if dialysis was necessary at the time of discharge. Gastrointestinal complications included gastrointestinal bleeding and visceral ischemia. Cardiac decompensation included New York Heart Classification classes III and IV.

### Statistical analysis

For comparison, the propensity score-matched groups were generated based on variables including demographics, co-morbidities and haemodynamic values. The patients were matched using a 1:1 nearest-neighbour algorithm. The statistical analyses were done using IBM SPSS, version 24 (IBM Corp., Armonk, NY, USA). Categorical data are described as percentages and continuous variables are presented as mean ± standard deviation or the median with the 25th and 75th quartiles. Statistical significance was defined as *p* ≤ 0.05. The mean values were compared using *t*-Student test for normally distributed variables and the Mann–Whitney *U*-test for variables that were not normally distributed. Cumulative survival was described using the Kaplan–Meier method. Categorical variables were compared using chi-square test or Fisher’s exact test.

**Table 1:** Baseline characteristics, propensity score-matched cohorts.

Characteristics	Unmatched	Sternotomy	Mini-MVS	*p* (Sternotomy vs mini-MVS)
*n*	669	227	227	
Age, years (median; 25th to 75th percentile)	64; 54–72	63.0	62.0	0.765
Male	447 (66.8%)	151 (66.5%)	153 (67.4%)	0.842
Body mass index, kg/m^2^ (median; 25th to 75th percentile)	25.4; 23.1–28	25.4	25.3	0.827
Diabetes	80 (12%)	22 (9.7%)	32 (14.1%)	0.419
Hyperlipidemia	284 (42.5%)	94 (41.4%)	107 (47.1%)	0.059
Arterial hypertension	490 (73.2%)	164 (72.2%)	162 (71.4%)	0.574
Pulmonary hypertension	299 (44.7%)	96 (42.3%)	100 (44.1%)	0.705
COPD: Chronic obstructive pulmonary disease	44 (6.6%)	12 (2.6%)	8 (1.8%)	0.36
GFR: Glomerular filtration rate < 60 ml/min	149 (22.4%)	71 (31.3%)	71 (31.3%)	0.999
Smoking	203 (30.3%)	69 (30.4%)	71 (31.3%)	0.393
Sinus rhythm	568 (84.9%)	193 (85.0%)	196 (86.3%)	0.688
Ejection fraction ≥50%	436 (65.9%)	154 (67.8%)	163 (72.1%)	0.513
Ejection fraction 31–50%	162 (24.2)	53 (23.3%)	50 (22.1%)	
Ejection fraction ≤ 30%	42 (6.3%)	10 (4.4%)	6 (2.7%)	
History of cardiac decompensation	171 (25.6%)	53 (23.3%)	46 (20.3%)	0.426
History of cardiopulmonary resuscitation	11 (1.6%)	6 (2.6%)	1 (0.4%)	0.098
Stroke preoperatively	22 (3.3%)	15 (6.6%)	3 (1.3%)	0.105
History of cardiac infarction	79 (11.8%)	26 (11.5%)	18 (7.9%)	0.265
Syncope	42 (6.3%)	14 (6.2%)	15 (6.6%)	0.596
History of percutaneous transluminal coronary angioplasty	66 (9.9%)	21 (9.3%)	14 (6.2%)	0.218

### Surgical technique

Our surgical approaches have been previously described in detail (Atluri et al., 2016; Glauber et al., 2015; Miceli et al., 2015). For the conventional MVS, mostly the standard median sternotomy with central bicaval cannulation was used. Briefly, the minimally invasive MVS was performed through a right anterolateral thoracotomy, mostly in the fourth intercostal space and femoral cannulation for cardio-pulmonary bypass was used. Various mitral valve repair techniques were performed. For example, we used ring annuloplasty, leaflet resection (quadrangular or triangular resection), neochordae and commissuroplasty. For the mitral valve replacement, we used the biological prosthesis Hancock II, Perimount Magna prostheses or mechanical St-Jude medical prostheses. In all cases transesophageal echocardiography was performed to evaluate the surgical results.

## Results

### Baseline characteristic

The median age of the patients in the matched-propensity analysis was 63 and 62 years in the mini-MVS and the conv-MVS group, respectively (*p* = 0.765). The male gender was predominant in both groups. The haemodynamic profiles of the matched patients were comparable in both groups. The demographic characteristics and mitral valve pathology grouped by Carpentier’s classification of the propensity-matched cohorts are outlined in detail in Tables 1 and 2.

**Table 2:** Mitral valve pathologies.

Carpentier-Classification	Unmatched	Sternotomy	Mini-thoracotomy	*p*
Only 1	27 (4%)	9 (4%)	10 (4.4%)	0.815
Only 2	119 (17.8%)	50 (22%)	39 (17.2%)	0.193
Only 3	22 (3.3%)	11(4.8%)	3 (1.3%)	0.03
Mixed	492 (73.5%)	156 (68.7%)	172 (75.8%)	0.09
Prosthetic valve	10 (1.5%)	1 (0.4%)	3 (1.3%)	0.315

### Clinical results

The in-hospital outcomes and complications are summarized in Table 3. In terms of in-hospital mortality, there was a significant difference between the investigated groups (mini-MVS: *n* = 1; 0.4% vs. *n* = 7; 3.1% in conv-MVS; *p* = 0.032). In the mini-MVS group, strokes occurred in 1.8% (*n* = 4) vs 0.9% (*n* = 2) in the conv-MVS group (*p* = 0.264). Furthermore, symptomatic transitory psychotic syndrome was observed in 28 patients (12.3%) in the mini-MVS group, which was similar to the incidence in the conv-MVS group (*n* = 23; 10.1%; *p* = 0.457). Moreover, there were two cases (0.9%) of myocardial infarctions after mini-MVS postoperatively, although these were without statistical significance in comparison with conv-MVS (*p* = 0.562). Exploration due to mediastinal bleeding did not differ significantly between the matched groups (*p* = 0.411). The transfusion requirements were significantly lower in the minimally invasive matched cohorts (*p* < 0.001). The incidence of postoperative respiratory failure requiring re-intubation (*p* = 0.647) or tracheotomy (*p* = 0.760) were comparable and did not differ significantly. Mini-MVS was at an advantage regarding acute renal failure with a necessity for dialysis (*n* = 6; 2.6% for mini-MVS vs. *n* = 15, 6.6% for conv-MVS; *p* = 0.044). The incidence of sepsis and pneumonia was comparable between the matched groups. The rate of pacemaker implantation did not differ between the analyzed groups (*p* = 0.177). There were one thoracic wound infection without necessity of surgical revision after the anterolateral thoracotomy, whereas one patient had severe wound infection requiring surgical revision in the conventional group (*p* = 1.000). The intensive care duration and the length of stay in hospital were comparable between the groups (*p* = 0.779 and *p* = 0.516), respectively. Postoperatively, both groups presented comparable median pain intensity rated on the visual analogue scale from 0 to 10: mini-MVS had a score 3.4 vs 3.5 for con-MVS (*p* = 0.862). The incidence of phrenic nerve palsy in both groups was comparable (*p* = 0.154).

**Table 3:** Postoperative outcomes, stratified by operative approach.

Characteristics	Unmatched	Sternotomy	Mini-MVS	*p* value
Mitral valve repair	397 (59.3%)	105 (46.3%)	184 (81.1%)	<0.0001
30-day mortality	14 (2.1%)	7 (3.1%)	1 (0.4%)	0.032
Intraoperative mortality	3 (0.4%)	0.0	1 (0.4%)	0.368
Stroke	6 (0.9%)	2 (0.9%)	4 (1.8%)	0.264
Re-exploration	10 (1.5%)	4 (1.8%)	2 (0.9%)	0.411
Dialysis	36 (5.4%)	15 (6.6%)	6 (2.6%)	0.044
Myocardial infarction	5 (0.7%)	1 (0.4%)	2 (0.9%)	0.562
Mediastinitis	0	0.0	0.0	
Wound infection	3 (0.4%)	1 (0.4%)	1 (0.4%)	1.000
Aortic dissection	0	0.0	0.0	
Re-intubation	27 (4%)	9 (4.0%)	11 (4.8%)	0.647
Tracheotomy	22 (3.2%)	6 (2.6%)	5 (2.2%)	0.760
Pacemaker implantation	10 (1.5%)	4 (1.8%)	1 (0.4%)	0.177
Atrial fibrillation	215 (32.1%)	71 (31.3%)	74 (32.6%)	0.321
Low cardiac output	50 (7.5%)	18 (7.9%)	8 (3.5%)	0.422
Transient psychotic syndrome	76 (11.4%)	23 (10.1%)	28 (12.3%)	0.457
Phrenic nerve palsy	8 (1.2%)	2 (0.9%)	6 (2.6%)	0.154
Sepsis	28 (4.2%)	12 (5.3%)	6 (2.6%)	0.149
Pneumonia	53 (7.9%)	19 (8.4%)	11 (4.8%)	0.131
Cardiopulmonary resuscitation	16 (2.4%)	8 (3.5%)	6 (2.6%)	0.587
Gastrointestinal complications	25 (3.7%)	10 (4.4%)	5 (2.2%)	0.189
Postoperative pain in subjective 0–10 scale, median; 25th to 75th percentile	3; 1–5	3; 1–5	3; 1–5	0.862
Intensive care unit length of stay [days], median; 25th to 75th percentile	1; 1–3	1; 1–3	1; 1–3	0.779
Hospital length of stay [days], median; 25th to 75th percentile	13; 9–18	12; 9–18	13; 10–17	0.516
Length of the mechanical ventilation [hours], median; 25th to 75th percentile	17; 13–22	18.89	22.76	0.184

### Surgical results

In the mini-MVS group the median operation time was 282 min and the CPB time was 178 vs. 200 min and 112 min in the conv-MVS cohort. These differences were statistically significant (*p* < 0.001). The cross-clamp times were significantly longer in the mini-MVS than in the sternotomy cohort (*p* < 0.001). Patients in the mini-MVS group received mitral valve repair more frequently (81.1% vs. 46.3%; *p* < 0.001) despite the same distribution of mitral valve apparatus defects (Table 2). No conversion to sternotomy was necessary and there was no case of aortic dissection in our collective. There were 218 patients (96.0%) who underwent mini-MVS and 12 patients (5.3%) who underwent conventional sternotomy using femoral arterial cannulation (*p* < 0.001); there was aortic cannulation in 18 cases (7.9%) for mini-MVS and in 214 cases (94.7%) for conventional sternotomy (*p* < 0.001).

Crystalloid cardioplegia was used in all patients in the mini-MVS cohort and in 224 in the conventional cohort. Blood cardioplegia was used only in three cases of the conventional cohort. Five patients (2.2%) in the mini-MVS cohort vs. 13 patients (5.7%) in the conv-MVS group received intra-aortic balloon pump (*p* = 0.018), and only one patient (0.4%) received extracorporeal membrane oxygenation in the conv-MVS group (*p* = 0.317). The surgical data are described in Table 4.

**Table 4:** Operative data.

Characteristics	Unmatched	Sternotomy	Mini-MVS	*p* value
Cardiopulmonary bypass time [min], (median; 25th to 75th percentile)	142; 107–187	112; 90–141	178; 148–211	<0.001
Cross-clamp time [min], (median; 25th to 75th percentile)	88; 64–114	73; 58–92	108; 89–129	<0.001
Aortic cannulation	363 (54.3)	214 (94.7%)	18 (7.9%)	<0.001
Femoral arterial cannulation	321 (48.1%)	12 (5.3%)	218 (96.0%)	<0.001
Red blood cell units, total (median; 25th to 75th percentile)	0; 0–2	1; 0–3	0; 0–2	0.001
Intra-aortic balloon pump	36 (5.4%)	13 (5.7%)	5 (2.2%)	0.018
Extracorporeal life support	1 (0.1%)	1 (0.4%)	0.0	0.317

### Follow-up results

**Figure 1:**
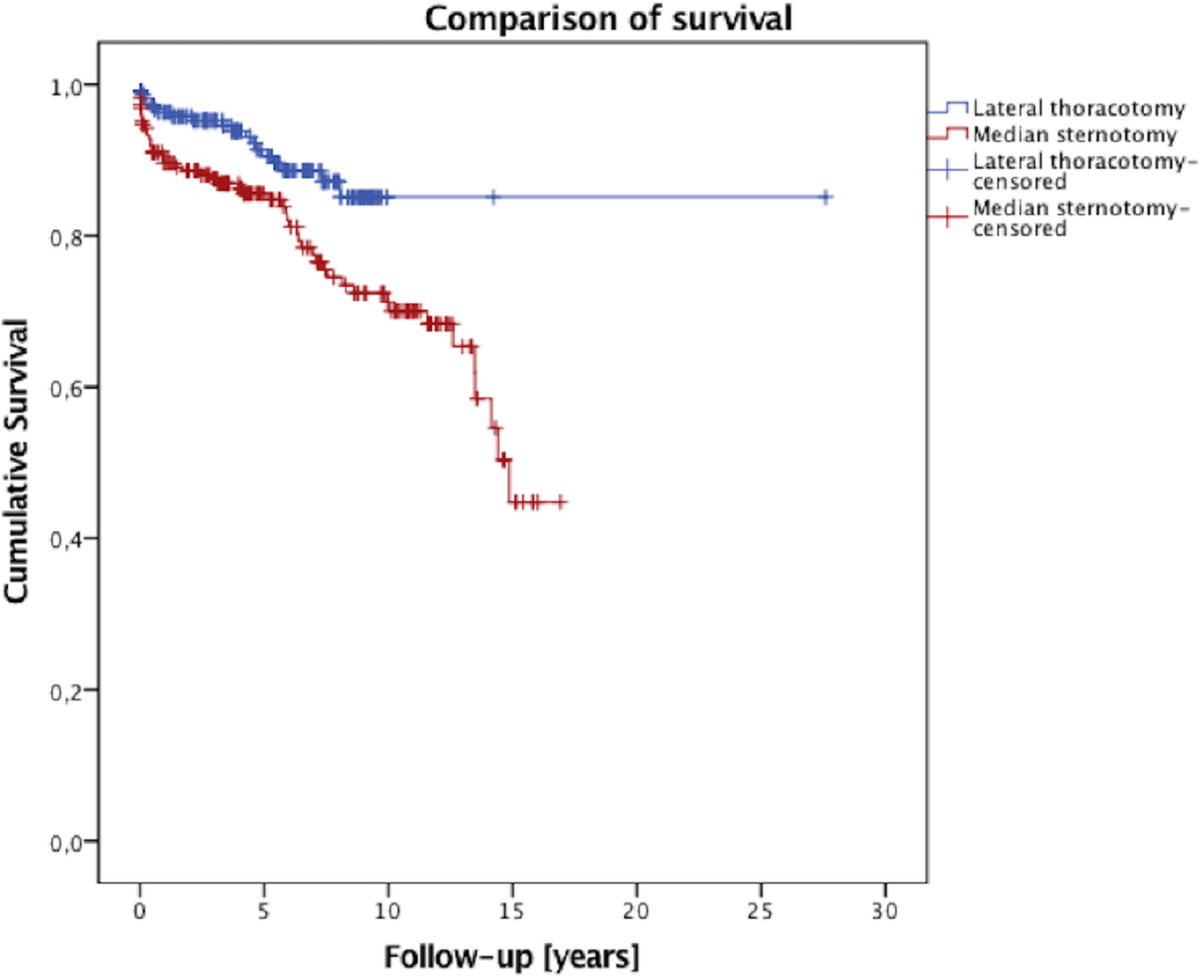
Survival curve.

The survival data are illustrated in Fig. 1. The median follow-up time was 4.4 years. One-, five-, and 10-year survival rates were 96%, 90%, and 84% after the minimally invasive approach, and 89%, 85%, and 70% after the conventional sternotomy, respectively (log rank *p* = 0.004, Breslow *p* = 0.004). In both the mini-MVS group and in the conv-MVS group there were two valve-related re-operations in each group, (*p* = 0.881). In the mini-MVS cohort the patients were operated on after 30 and 72 months and in the cohort of the conventional approach after 24 and 38 months. At the final follow-up interview, 51 patients (50.5%) were presented at New York Heart Association (NYHA) Classes I and II after minimally invasive surgery which is correspondingly comparable with the conv-MVS group (50.6%, *n* = 44; *p* = 0.727). Furthermore, both groups did not differ with regard to exercise capacity (*p* = 0.153). During the follow-up time, five patients had strokes in the mini-MVS cohort, while no stroke occurred in the conv-MVS cohort, (*p* = 0.036). However, no patient suffered from myocardial infarction in the mini-MVS group, although two patients in the conv-MVS group had myocardial infarction in the follow-up period, (*p* = 0.124). The patients’ scar satisfaction regarding the cosmetic results was comparable in the matched groups (*p* = 0.771). The follow-up results are summarized in Table 5.

**Table 5:** Follow-up data.

Characteristics	Unmatched	Sternotomy	Mini-MVS	*p* value
Median follow-up (years; 25th to 75th percentile)	4.2; 1.8–7.7	4.45; 1.96–10.24	4.44; 2.15–7.4	0.064
Answered to questionnaire	189	87	102	
During follow-up:				
Myocardial infarction	2 (1%)	2 (2.3%)	0.0	0.124
Stroke	5 (2.6%)	0.0	5 (4.9%)	0.036
Mitral valve replacement after repair	4 (2.1%)	2 (2.3%)	2 (2.0%)	0.881
Pacemaker implantation	2 (1%)	1 (1.1%)	1 (1.0%)	0.915
Implantable cardioverter defibrillator implantation	2 (1%)	2 (2.3%)	0.0	0.126
Heart transplantation	1 (0.6%)	0.0	1 (1.0%)	0.352
Congestive heart failure				
NYHA class I or less	84 (44.4%)	40 (45.9%)	45 (44.1%)	0.727
NYHA class II	72 (38%)	34 (39.1%)	38 (37.6%)	
NYHA class III	30 (15.9%)	11 (12.6%)	19 (18.8%)	
NYHA class IV	3 (1.6%)	2 (2.3%)	1 (1.0%)	
Exercise capacity				
Good	145 (76.7%)	63 (72.4%)	82 (81.2%)	0.153
Reduced	43 (22.8%)	24 (27.6%)	19 (18.8%)	
Satisfaction with cosmetic results	174 (92%)	80 (92.0%)	94 (93.1%)	0.771
One-year survival	93%	89%	96%	0.004
Five-year survival	85%	85%	90%	
Ten-year survival	71%	70%	84%	

## Discussion

In our present study, we showed significantly lower early mortality with better long-term survival rates in the mini-MVS cohort. Recent series were also able to demonstrate, that mini-MVS can be performed without significant differences in mortality and long-term survival compared with conventional MVS (Cheng et al., 2011; Goldstone et al., 2013; Moscarelli et al., 2016a; Sundermann, Czerny & Falk, 2015). The elevated stroke rate after mini-MVS as a consequence of peripheral cannulation or retrograde perfusion is considered to be disadvantage of this approach (Bedeir et al., 2015). However, some newer publications disprove these findings. Several studies showed no significant differences regarding the incidence of stroke in comparison to both surgical approaches (Atluri et al., 2016; Downs et al., 2016; Goldstone et al., 2013). Modi et al. reported a systemic meta-analysis of six studies, that showed similar stroke rates between mini-MVS and the conventional method (Modi, Hassan & Chitwood, 2008). In our propensity score-matched analysis, there were also no differences in stroke rates between patients undergoing mini-MVS and conventional sternotomy in the immediate postoperative period. Surprisingly, the mini-MVS cohort developed more strokes in the long-term follow-up than did the conventional cohort. Our follow-up data did not allow us information about whether these strokes are complications of valve-related anticoagulation.

Furthermore, we observed prolonged CPB and cross-clamp times in the mini-MVS cohort. These facts did not influence the early mortality. In contrast, patients undergoing the mini-MVS approach suffered significantly less from renal failure requiring dialysis and showed better early survival. Other authors underline these findings (Atluri et al., 2016; Cheng et al., 2011; Downs et al., 2016; Goldstone et al., 2013; Sundermann, Czerny & Falk, 2015). Bleeding events, respiratory failure, and wound infection frequencies are comparable with the results of conv-MVS. Similar results were achieved in other contemporary series (Atluri et al., 2016; Downs et al., 2016; Goldstone et al., 2013; Sundermann, Czerny & Falk, 2015).

The indication for mini-MVS should be well thought out. Not all patients with isolated mitral valve disease are ideal candidates for this approach. Contraindications are very poor left ventricular ejection fraction, obesity, serious lung disease, chest wall depth, or pleural adhesions (Glauber et al., 2015; Ward, Grossi & Galloway, 2013). All of these factors must be carefully examined in decision making. In our collective, conversion to conventional sternotomy was not necessary in any case. One of the main aims of minimally invasive surgery is to improve cosmetic results and reduce pain intensity. Cheng et al. showed in a meta-analysis improved patient’s scar satisfaction, meanwhile, Svensson and colleagues showed less pain intensity within the first 24 h postoperatively after the minimally invasive approach (Cheng et al., 2011; Svensson et al., 2010). Our matched cohorts showed high satisfaction with the cosmetic results and comparable pain intensity postoperatively in both groups. Previous series examined the influence of minimally invasive surgery on the success of mitral valve repair; it led to similar or superior repair rates. We achieved a remarkably higher mitral valve repair rate among the mini-MVS cohort with 81.1% vs. 46.3% through the conventional approach (Downs et al., 2016; Moscarelli et al., 2016b; Sundermann, Czerny & Falk, 2015). During the follow-up time, the incidence of myocardial infarction was low and comparable in both matched groups. However, the mini-MVS cohort showed significantly better long-term survival. Most analyses showed comparable long-term survival after the mini-MVS (Goldstone et al., 2013; Iribarne et al., 2012; Moscarelli et al., 2016a; Svensson et al., 2010). With regard to mitral valve-related re-intervention, similar results were achieved in our comparison. Recent literature underlines our findings (Downs et al., 2016; Galloway et al., 2009; Sundermann, Czerny & Falk, 2015).

## Study Limitation

The main limitation of our study is its retrospective design; however the follow-up data were obtained prospectively. To our knowledge, this is one of few contemporary propensity score-matched comparisons of MVS that is stratified by operative approaches. Nevertheless, the results of our non-high volume center for MVS are comparable with high-volume centers for MVS. We demonstrated excellent results with a median follow-up time of 4.4 years after mini-MVS. However, the lack of echocardiographic data is an important limiting factor of the follow-up. Furthermore, our data are limited by the lack of consideration of surgeon expertise and operation date in the matching score analysis. However, the expertise of surgeons may have changed over this extended period and the repair technique of mitral valve pathologies have also changed. Furthermore, the better survival after mini-MVS could be translated as a result of higher repair rates in this collective.

## Conclusion

Despite the longer operative times, mini-MVS can be considered a safe and equivalent alternative to conventional sternotomy. We were able to show superior short- and long-term survival after mini-MVS. We also showed that mini-MVS leads to higher repair rates with excellent long-term outcomes. One of the most important points in our comparison was to eliminate the preoperative differences with the equal distribution of mitral valve defects. Also in the setting, the minimally invasive approach was superior with regard to the repair rates. Minimally invasive valve surgery offers various advantages over the conventional approach. To compete with rapidly developing percutaneous technologies, these results should encourage us to offer this technique even to elderly patients. In summary, mini-MVS should be preferred if there is a suitable anatomy of the mitral valve and the patient’s conditions enable this approach. Furthermore, well-thought-out patient selection is very important for a successful outcome.

## Supplemental Information

10.7717/peerj.4810/supp-1 Supplemental Information 1 Raw data.

## Additional Information and Declarations

### Data Availability

The following information was supplied regarding data availability: The raw data are provided in a Supplemental File.
